# INTEGRATING TELEHEALTH AND COMMUNITY HEALTH WORKERS TO ENHANCE QUALITY CARE ACCESS: A NARRATIVE REVIEW

**DOI:** 10.1093/geroni/igac059.2465

**Published:** 2022-12-20

**Authors:** Ilana Engel, Alice Zhang, Robert Stiles, Eve-Lynn Nelson, Amber Watts

**Affiliations:** University of Kansas, Lawrence, Kansas, United States; University of Kansas Medical Center, Kansas City, Kansas, United States; University of Kansas Medical Center, Kansas City, Kansas, United States; University of Kansas Medical Center, Kansas City, Kansas, United States; University of Kansas, Lawrence, Kansas, United States

## Abstract

Community Health Workers (CHWs) often share cultural, geographic, or other lived experiences with patients and provide health education and support. Use of CHWs and telehealth approaches are promising strategies for addressing the needs of patients with metabolic syndrome (MetS). This narrative review analyzed how these approaches were integrated into programs expanding care access for patients with MetS. Searching PubMed, PSYCInfo, Embase, Web of Science, and Google Scholar resulted in 1,630+ abstracts screened and 12 articles meeting inclusion criteria. These studies examined implementation of tele-mentoring approaches (n=4), patient group classes via videoconferencing (n=2), or individual telehealth consultations facilitated by CHWs (n=7), with some programs including multiple intervention types. This review included adults ranging from 37-79 years old. Most studies focused on late mid-life (ages 50-64). Because health behaviors in midlife have important implications for MetS and related health concerns in later life, it is important to consider midlife interventions. Using the RE-AIM framework, we evaluated studies on five dimensions: reach, effectiveness, adoption, implementation, and maintenance. Reach and implementation indicators suggest reducing barriers to engagement (e.g., home visits) allows for higher participation and program completion rates. Measures of MetS-related behavioral outcomes were heterogeneous across study designs, making overall effectiveness difficult to determine. Adjusting time spent with patients according to health literacy and clinical needs is a strategy CHW programs use to provide equitable, cost-effective care. Programmatic considerations for implementing programs that include both CHWs and telehealth are discussed, with special consideration for what works in late middle age and in older adulthood.

